# Micro- and Macronutrient Intake in Elderly Costa Ricans: The Costa Rican Longevity and Healthy Aging Study (CRELES)

**DOI:** 10.3390/nu15061446

**Published:** 2023-03-17

**Authors:** Shengxin Yu, Ana Baylin, Edward A. Ruiz-Narváez

**Affiliations:** 1 Department of Nutritional Sciences, University of Michigan School of Public Health, Ann Arbor, MI 48109, USA; 2 Department of Epidemiology, University of Michigan School of Public Health, Ann Arbor, MI 48109, USA

**Keywords:** longevity, aging, diet, macronutrients, micronutrients, Costa Rica

## Abstract

Costa Rica, a middle-income country in Central America, has a life expectancy similar or even higher than richer countries. This survival advantage is more evident among the elderly, who have one of the lowest mortality rates in the world. Dietary factors may play a role in this extended longevity. We have shown that a traditional rural diet is associated with longer leukocyte telomere length—a biomarker of aging—among elderly Costa Ricans. In the present study, we used data from the Costa Rican Longevity and Healthy Aging Study (CRELES) to characterize further rural and urban diets of the elderly (60+ years). A validated food frequency questionnaire was used to assess usual diet. We used energy-adjusted regression models to compare the intake of micro- and macronutrients between rural and urban regions of the country. Elderly rural residents had a higher consumption of carbohydrates (but lower glycemic index), fiber, dietary iron, and used more palm oil for cooking than elderly urban dwellers. On the other hand, elderly subjects living in urban areas had a higher intake of total fat, mono and polyunsaturated fat, alcohol and dietary calcium compared to elderly rural residents. Our results are similar to earlier reports of middle-aged Costa Ricans and add to the characterization of diet differences in rural and urban regions of the country.

## 1. Introduction

Costa Rica, a middle-income country in Central America, has a life expectancy at birth similar to high-income countries and even higher than the US [1]. Extended longevity is more evident in elderly Costa Rican men, whose mortality rate is among the lowest in the world [2,3]. Among a diversity of factors [1,4], diet may play a key role on the observed lower mortality in elderly Costa Ricans. We have recently shown that a traditional diet, which is more frequent in rural parts of the country, was associated with longer leukocyte telomere length (LTL)—a biomarker of biologic aging—in elderly Costa Ricans [5]. Another study found that diets in rural parts of Costa Rica are richer in fiber and lower in total fat as compared to urban diets [6]. However, this last study was among middle-aged subjects (mean age = 57 years old), and it was geographically restricted to the capital city San José and surrounding counties. It remains to be examined how rural and urban diets differ among elderly Costa Ricans across the whole country.

In the present study, we used data from the Costa Rican Longevity and Healthy Aging Study (CRELES) to characterize further the diets of the elderly in rural and urban regions of the country. Because CRELES is a nationally representative study of the elderly Costa Rican population, our results would add relevant information about nutrient consumption among elderly Costa Ricans, a population group with a lower-than-expected mortality rate.

## 2. Methods

### 2.1. Study Population

We used publicly available and de-identified data from the Costa Rican Longevity and Healthy Study (CRELES) to carry out all analyses in the present work. CRELES is a nationally representative longitudinal survey of health and life course experiences of nearly 3000 elderly Costa Ricans ages 60 and older in 2005 [7]. CRELES is carried out by the Central America Population Center at the University of Costa Rica. CRELES objectives are to examine determinants of life expectancy and healthy aging in the Costa Rican elderly population. Participants were selected using the 2000 population census among those who were born in 1945 or before, with an over-sample of the oldest elderly people (ages 95 and over). Costa Rica has a universal public health care system that is administered by the autonomous Costa Rican Social Security Fund known as CCSS (Spanish acronym for “Caja Costarricense de Seguro Social”). The CCSS divides the Costa Rica territory into 102 first-level health areas. CRELES took a systematic sample of 60 health areas, covering 59% of the country’s territory.

### 2.2. Data Collection

Trained personnel carried out data collection in three waves of household interviews. The baseline interview was conducted between November 2004 and September 2006. Participants were asked a series of questions about health status, living conditions, history of some diseases, socioeconomic status, and food intake. More than 90% of the participants provided blood and urine samples. Questionnaire data were updated in two additional rounds of interviews: from October 2006 to July 2008 among 2364 surviving participants; and from February 2009 to January 2010 among 1855 surviving participants. All de-identified CRELES databases from waves 1, 2, and 3 are public and available from The National Archive of Computerized Data on Aging of the University of Michigan: http://www.icpsr.umich.edu/icpsrweb/NACDA/series/386 (accessed on 25 July 2019).

### 2.3. Dietary Information

The dietary information of CRELES participants was collected using a modified and abbreviated version (27 food items) of a food-frequency questionnaire (FFQ) that was developed and validated to use in the adult population of Costa Rica [8,9]. The reduced FFQ that was used in CRELES explains more than 75% of the variance of the selected macro- and micronutrients (e.g., 81% of total energy intake, 85% of total fat, 80% of protein, 76% of carbohydrates, and 76% of dietary fiber) [5].

### 2.4. Statistical Analysis

All analyses are based on data from the 2819 CRELES participants who provided dietary information in the baseline interview. Nutrient intakes were adjusted for total caloric intake using the methods of residuals [10]. We calculated weighted descriptive statistics of CRELES participants stratifying by place of residence (rural vs. urban). We used two-tailed t-tests to examine the difference in means for continuous variables, and chi-square tests to evaluate the difference in frequency distributions for categorical variables. We ran age- and sex-adjusted regression models to assess whether mean intakes of energy-adjusted macro- and micronutrients are different between rural and urban regions of Costa Rica. We also conducted sex-stratified analyses to evaluate whether rural vs. urban differences are the same regardless of sex. We used SAS software version 9.4 (SAS Institute, Cary, NC, USA) to carry out all statistical analyses.

## 3. Results

Table 1 shows the descriptive characteristics of rural and urban populations of elderly Costa Ricans. Compared to the urban parts of the country, rural regions were characterized with lower proportions of women, high-school graduates, and alcohol drinkers. The mean BMI and total plasma cholesterol levels were also lower in elderly rural populations. On the other hand, smoking prevalence was higher in rural regions.

Table 2 shows energy-adjusted nutrient intakes and use of different types of cooking oil by rural and urbans regions of Costa Rica. Elderly rural residents reported significantly lower intakes of total fat, monounsaturated fat (MUFA), n-3 and n-6 polyunsaturated fat (PUFA), alcohol, calcium, and the use of multivitamins compared to those living in urban areas. The consumption of carbohydrates was higher among rural elderly dwellers, but their mean glycemic index was lower relative to elderly urban residents. Dietary iron, excluding supplements, and fiber were higher in rural regions. Regarding types of cooking oil, elderly residents in rural regions used more palm oil and less soybean oil compared to elderly residents in urban areas of the country. 

Table 3 shows sex-stratified comparisons between rural and urban regions. Overall, we observed the same results as the ones without stratification. The only difference was the intake of saturated fat. Even though total fat consumption was lower in rural regions regardless of sex, the intake of saturated fat was higher among elderly women living in rural areas compared to elderly women in urban regions. There was no significant difference in saturated fat consumption between rural and urban elderly men.

## 4. Discussion

In the present work, we described and compared intakes of different macro- and micronutrients of elderly subjects living in rural and urban regions of Costa Rica. Elderly Costa Ricans, particularly in rural areas, have one of the lowest mortality rates in the world [2,3]. We also evaluated differences on the prevalence of cardiovascular disease (CVD) risk factors. Rural parts of the country, compared to urban areas, had a mix of protective and high-risk CVD factors. For example, mean BMI and plasma total-cholesterol levels were lower among elderly rural residents but smoking prevalence was higher. The observed lower levels of total plasma cholesterol in elderly rural residents despite the higher use of cooking saturated-fat-rich palm oil in rural areas is noteworthy. However, as we discuss below in more detail, elderly rural Costa Ricans also consume more dietary fiber and low-glycemic carbohydrates than their urban counterparts do. These healthy foods may outweigh the harmful effects of palm oil on total plasma cholesterol levels. We did not observe any significant difference for other CVD risk factors such as regular physical activity, prevalence of diabetes and hypertension, and plasma levels of HDL cholesterol, LDL cholesterol, and triglycerides.

Regarding dietary behavior, there were several noteworthy differences. Total caloric intake was lower in rural vs. urban areas and the difference was driven by lower energy consumption among rural elderly women compared to urban elderly women. Men consumed a similar number of calories regardless of their place of residence (rural vs. urban). The consumption of total fat as well as MUFA, n-3 and n-6 PUFA was lower in rural areas compared to urban regions. Our data, as well as previous results from middle-aged Costa Ricans [6], showed that the use of soybean cooking oil—probably indicating lower socioeconomic status [11]—was lower in rural areas; this may explain the low consumption of PUFA and low plasma cholesterol in these communities.

The sex-stratified analysis showed that rural elderly women had a higher intake of saturated fat than their urban counterparts. No such difference was observed among elderly men. Higher saturated fat intake in elderly rural women could be due in part to their higher use of saturated-fat-rich palm oil. The use of palm oil, which is rich in saturated fat [12], was higher in rural regions of the country, and the difference of use (rural vs. urban) was more pronounced among women. Elderly rural women used palm oil more than twice compared to elderly urban women (16.8% vs. 7.0%, respectively). The prevalence of use of palm oil among elderly rural men was just 50% higher relative to the use among elderly urban men (13.6% vs. 8.8%, respectively). The high consumption of palm oil has been found associated with an increased risk of non-fatal myocardial infarction in middle-aged Costa Ricans [13]. The observed sex-specific rural vs. urban differences on the use of palm oil for cooking suggest that, among elderly Costa Ricans, palm oil is less of a cardiovascular risk factor when comparing rural men vs. urban men than for rural women vs. their urban counterparts.

It is noteworthy that even though carbohydrate intake was higher in rural regions, their mean dietary glycemic index was lower compared to urban areas. We also found that the intake of fiber was higher among elderly rural residents, which is consistent with earlier reports from middle-aged Costa Ricans [6]. A higher fiber intake in rural communities is explained by their higher consumption of beans [14], a major source of dietary fiber in the Costa Rican diet [15]. We have recently shown that a traditional rural dietary pattern rich in bean consumption was more prevalent among elderly rural residents compared to their urban counterparts, and was associated with longer leukocyte telomere length, a biomarker of biologic aging [5]. This traditional rural dietary pattern includes a mix of beneficial and harmful foods. For example, the pattern is rich in fiber and low in glycemic index but high in the use of palm oil. However, when put together, the healthy components seem to outweigh the unhealthy foods, as shown by the lower total plasma cholesterol in elderly rural Costa Ricans compared to urban dwellers in the present study, and the previously reported association of this traditional rural dietary pattern with longer leukocyte telomeres [5].

Our study has several strengths. First, CRELES is a nationally representative study of the population of elderly Costa Ricans, making our results generalizable to this target group. Second, the relatively large sample size allowed for us to obtain precise estimates of the mean consumption of nutrients of interest. We notice some limitations too. The abbreviated FFQ (27 food items) may fail to capture relevant nutrient variation. However, as we have previously reported [5], this reduced FFQ is able to capture most (75% or higher) of the variation in micro- and macronutrient intake. In addition, we have been able to identify major dietary patterns using the CRELES FFQ [5], showing that we are capturing relevant food intake variation.

In summary, the current report adds to the characterization of the diet of elderly Costa Ricans and how they may be different by their place of residence (rural vs. urban). Overall, our results are consistent with early data from middle-aged Costa Ricans [6], showing that rural diets are characterized by a dietary pattern consisting of a lower fat and higher fiber intake, lower glycemic index, and more use of palm oil for cooking as compared to urban diets. We also found that elderly rural residents had a lower mean BMI and total plasma cholesterol compared to their urban counterparts. Lower plasma total cholesterol may be due in part to the healthy components (e.g., high dietary fiber and low glycemic index foods) of the traditional rural dietary pattern. This may explain, in part, the lower mortality that is observed among older Costa Ricans living in certain rural areas relative to elderly urban residents [2,3].

## Figures and Tables

**Table 1:** Characteristics of CRELES participants: overall and according to place of residence.

Characteristic	Mean ± SD, %			
	Rural	N	Urban	N
Age, years	70.3 ± 7.7	1123	70.5 ± 8.4	1696
Women, % ^a^	46.4	1123	56.2	1696
Education 11+ years, % ^a^	4.5	1123	17.2	1696
Body mass index, kg/m^2 a^	26.2 ± 5.1	1084	27.3 ± 5.3	1612
Regular exercise, %	32.4	1123	30.8	1690
Current smokers, % ^a^	12.1	1118	8.7	1687
Current alcohol drinkers, % ^a^	31.0	1119	34.2	1685
History of diabetes, %	31.2	1078	34.6	1587
History of hypertension, %	57.6	1118	61.0	1678
Plasma lipids, mg/dl				
Total cholesterol ^a^	213.1 ± 48.5	1075	217.0 ± 50.2	1579
HDL cholesterol	43.8 ± 12.5	1075	44.5 ± 13.6	1576
LDL cholesterol	136.7 ± 39.2	1016	139.8 ± 42.4	1481
Triacylglycerol	163.4 ± 88.5	1074	165.1 ± 94.6	1579

SD: standard deviation; HDL: high-density lipoprotein; LDL: low-density lipoprotein. ^a^ *p*-value < 0.05 comparing rural vs. urban.

**Table 2:** Comparison of energy-adjusted nutrient intakes and type of cooking oil used by elderly Costa Ricans living in rural and urban regions.

Nutrient	Age- and Sex-Adjusted Least Square Means (95% CI), %	
	Rural (n = 1123)	Urban (n = 1696)
Total energy, kcal/day ^a^	2071 (2030, 2112)	2130 (2096, 2163)
Protein, g/day	68.4 (67.9, 68.9)	68.6 (68.2, 69.1)
Total fat, g/day ^a^	69.3 (68.7, 70.0)	72.6 (72.0, 73.1)
Saturated fat, g/day	26.6 (26.1, 27.0)	26.0 (25.7, 26.4)
MUFA, g/day ^a^	22.5 (22.0, 22.9)	24.4 (24.0, 24.8)
n-3 PUFA, g/day ^a^	1.8 (1.7, 1.9)	2.0 (1.9, 2.1)
n-6 PUFA, g/day ^a^	15.2 (14.8, 15.5)	16.6 (16.3, 16.9)
*Trans* fatty acids, g/day	2.8 (2.7, 2.9)	2.9 (2.8, 3.0)
Total carbohydrate, g/day ^a^	305.5 (303.7, 307.3)	298.0 (296.6, 299.5)
Dietary glycemic index ^a^	75.6 (75.4, 75.8)	76.1 (75.9, 76.3)
Dietary fiber, g/day ^a^	23.2 (22.9, 23.5)	22.3 (22.1, 22.5)
Alcohol, g/day ^a,b^	1.7 (1.3, 2.1)	2.3 (1.9, 2.6)
Dietary cholesterol, mg/day	244.4 (238.0, 250.8)	249.3 (244.0, 254.5)
Use of multivitamins, % ^a^	4.9	8.4
Iron, mg/day ^a,c^	16.2 (16.1, 16.3)	16.0 (15.9, 16.1)
Calcium, mg/day ^a,c^	736.0 (718.8, 753.2)	788.0 (773.6, 802.4)
α-Tocopherol, mg/day ^c^	12.6 (11.6, 13.6)	13.1 (12.3, 13.9)
γ-Tocopherol, mg/day ^c^	7.4 (7.2, 7.6)	7.4 (7.2, 7.6)
Type of cooking oil, % ^a^		
Palm	15.2	7.7
Soybean	50.4	55.8
Other	34.5	36.5

SD: standard deviation; MUFA: monounsaturated fatty acids; PUFA: polyunsaturated fatty acids. ^a^ *p*-value < 0.05 comparing rural vs. urban regions. ^b^ Restricted to current alcohol drinkers. ^c^ Restricted to non-users of supplements.

**Table 3:** Sex-stratified comparison of total energy-adjusted macronutrient intakes and type of cooking oil used in rural and urban populations.

Nutrient	Age-Adjusted Least Square Means (95% CI), %			
	Female		Male	
	Rural (n = 543)	Urban (n = 988)	Rural (n = 580)	Urban (n = 708)
Total energy, kcal/day	1881.6 (1827.3, 1935.9) ^a^	2001.0 (1960.8, 2041.3)	2258.1 (2196.4, 2319.7)	2249.4 (2193.6, 2305.2)
Protein, g/day	68.8 (68.1, 69.5)	69.1 (68.6, 69.7)	68.1 (67.4, 68.8)	68.1 (67.5, 68.8)
Total fat, g/day	69.7 (68.8, 70.7) ^a^	72.9 (72.2, 73.6)	68.9 (68.0, 69.9) ^a^	72.3 (71.5, 73.2)
Saturated fat, g/day	27.2 (26.5, 27.9) ^a^	26.2 (25.7, 26.7)	26.0 (25.4, 26.6)	25.9 (25.4, 26.5)
MUFA, g/day	22.6 (22.0, 23.3) ^a^	24.5 (24.0, 25.0)	22.3 (21.6, 22.9) ^a^	24.3 (23.7, 24.9)
n-3 PUFA, g/day	1.8 (1.8, 1.9) ^a^	2.0 (1.9, 2.0)	1.8 (1.7, 1.8) ^a^	1.9 (1.9, 2.0)
n-6 PUFA, g/day	15.0 (14.5, 15.5) ^a^	16.7 (16.3, 17.1)	15.3 (14.9, 15.7) ^a^	16.4 (16.0, 16.8)
*Trans* fatty acids, g/day	2.8 (2.7, 2.9)	2.9 (2.8, 3.0)	2.7 (2.6, 2.8)	2.8 (2.7, 2.9)
Total carbohydrates, g/day	305.3 (302.8, 307.8) ^a^	299.0 (297.2, 300.9)	305.7 (303.1, 308.3) ^a^	296.8 (294.5, 299.2)
Dietary glycemic index	75.4 (75.2, 75.7) ^a^	76.1 (75.9, 76.3)	75.7 (75.4, 76.0) ^a^	76.1 (75.9, 76.4)
Dietary fiber, g/day	23.3 (23.0, 23.7) ^a^	22.4 (22.1, 22.7)	23.1 (22.7, 23.5) ^a^	22.1 (21.8, 22.5)
Alcohol, g/day ^b^	0.84 (0.59, 1.09)	0.95 (0.78, 1.13)	2.58 (1.77, 3.39)	3.65 (2.93, 4.36)
Dietary cholesterol, mg/day	233.6 (224.8, 242.5)	241.6 (235.1, 248.1)	255.0 (245.6, 264.3)	256.5 (248.0, 264.9)
Use of multivitamins, %	6.0 ^a^	10.8	3.7	5.8
Iron, mg/day ^c^	16.3 (16.2, 16.5) ^a^	16.1 (16.0, 16.2)	16.2 (16.0, 16.3) ^a^	15.9 (15.7, 16.0)
Calcium, mg/day ^c^	761.4 (734.4, 788.4) ^a^	840.4 (819.8, 861.0)	709.0 (687.8, 730.3)	732.4 (713.0, 751.9)
α-Tocopherol, mg/day ^c^	12.9 (11.4, 14.4)	13.4 (12.3, 14.6)	12.3 (11.1, 13.5)	12.8 (11.7, 13.9)
γ-Tocopherol, mg/day ^c^	7.3 (7.0, 7.6)	7.4 (7.1, 7.6)	7.5 (7.3, 7.8)	7.4 (7.2, 7.7)
Type of cooking oil, %				
Palm	16.8 ^a^	7.0	13.6 ^a^	8.8
Soybean	51.6 ^a^	56.3	49.2 ^a^	55.5
Other	31.6 ^a^	36.8	37.3 ^a^	35.7

SD: standard deviation; MUFA: monounsaturated fatty acids; PUFA: polyunsaturated fatty acids. ^a^ *p*-value < 0.05 comparing rural vs. urban regions. ^b^ Restricted to current alcohol drinkers. ^c^ Restricted to non-users of supplements.

## Data Availability

The data presented in this study are openly available in The National. Archive of Computerized Data on Aging of the University of Michigan: http://www.icpsr.umich.edu/icpsrweb/NACDA/series/386 (accessed on 25 July 2019).
